# Landscape

**DOI:** 10.3201/eid1107.AD1107

**Published:** 2005-07

**Authors:** Mary Oliver

**Keywords:** landscape, Mary Oliver, Poem

Isn't it plain the sheets of moss, except that
they have no tongues, could lecture
all day if they wanted about
spiritual patience? Isn't it clear
the black oaks along the path are standing
as though they were the most fragile of flowers?
Every morning I walk like this around
the pond, thinking: if the doors of my heart
ever close, I am as good as dead.
Every morning, so far, I'm alive. And now
the crows break off from the rest of the darkness
and burst up into the sky—as though
all night they had thought of what they would like
their lives to be, and imagined
their strong, thick wings.

**From Dream Work, copyright 1986 by Mary Oliver.**

Used by permission of Grove/Atlantic, Inc., and the author.

